# Research on the Robustness of the Chinese Input–Output Network Based on Relative Entropy Theory

**DOI:** 10.3390/e24081043

**Published:** 2022-07-29

**Authors:** Weidong Li, Anjian Wang, Wanli Xing

**Affiliations:** 1 Institute of Mineral Resources, Chinese Academy of Geological Sciences, Beijing 100037, China; lwd@email.cugb.edu.cn; 2 Research Center for Strategy of Global Mineral Resources, Chinese Academy of Geological Sciences, Beijing 100037, China; 3 Development and Research Center, Chinese Geological Survey, Beijing 100037, China

**Keywords:** input–output network, robustness, relative entropy, degree, strongest path

## Abstract

The input–output (IO) network is the quantitative description of an IO-based economy in which nodes represent industries and edges connecting nodes represent the economic connection between industries. Robustness refers to the ability of tolerating perturbations that might affect the system’s functional body. There is both practical and theoretical significance to explore the robustness of the IO network for economic development. In this paper, we probe the robustness of the Chinese IO network based on the relative entropy of the probability distribution of network parameters (node degree, strongest path betweenness, downstream closeness and upstream closeness) under random node or edge failure and intentional node or edge attack. It is found that the Chinese IO network shows relatively weak robustness when it is under intentional attack, but relatively strong robustness when it is under random failure. Our experiment also verifies the applicability and effectiveness of the relative entropy model in measuring the robustness of the IO network.

## 1. Introduction

Industrial relation is the basic relation in economic activity. With the rapid development of economic globalization and network information technology, the dependence and restriction relationship between industries has strengthened, and the interaction and division of labor among industries have played an increasingly prominent role in regional economic development. The input–output model established by Leontief [1] is a basic method to describe industrial correlation and is committed to quantitatively measuring the correlation between different industries.

The national economy is regarded as an organic whole in input–output analysis, in which a series of industrial sectors are mapped into a crisscrossed input–output table according to the input–output relationship in a certain period. Then, scholars can comprehensively study the quantitative relationship between each specific sector and make an economic analysis and prediction based on the input–output table. In essence, the intermediate matrix of the input–output table is a complex network in the sense that industries as nodes are linked with the exchange of products between industries whose structure can be characterized by power–law distributions or similar ones [2,3,4,5,6]. The complex network theory is employed by a large number of scholars to detect key industries [7,8] and industrial communities [9,10] and probe the risks of the transmission mechanism [11,12] in the input–output system.

Robustness is an important feature of complex networks which describes the ability to maintain structural integrity and function in the case of random failure or intentional attack for nodes or edges. Initially, Albert et al. [13] compared the robustness characteristics of random networks and scale-free networks under node attack through analogue simulation. Holme et al. [14] further comprehensively summarized that there are four main types of intentional attack against complex networks, namely, “ID removal”, “RD removal”, “IB removal” and “RB removal”, which are based on different attack strategies, initial degree distribution, initial betweenness, recalculated degree distribution and recalculated betweenness. Subsequently, research on robustness for the complex network is widely applied to the power network [15,16], biological molecular network [17] and trade network [18,19]. At present, studies on robustness are mainly focused on robustness measures [14,20,21] and robust control [15,17,19]. The former mainly quantitatively measures robustness by using various indicators to solve the problem of “which network has better robustness”, while the latter mainly tends to improve the network robustness by designing reasonable and effective measures, so as to achieve the purpose of controlling network robustness.

Information entropy, first proposed by Shannon, is used to measure the randomness of a system. The bigger the information entropy is, the more chaotic the system is, and vice versa. As an important branch of information entropy, relative entropy is a new concept developed by Kullback and Leibler [22] on the basis of information entropy. It is powerful in measuring the distance or similarity between two random distributions, and has been applied to hierarchical policy search by many scholars [23,24], key nodes identification [25,26] and node similarity measurement [27,28].

However, distinguished from other complex networks, the current research on the robustness of the input–output network is relatively weak. For example, while the “fragility” of the global input–output network mentioned by Grazzini and Spelta [12] is similar to its robustness, there are no relevant experiments under random failure or intentional attack that systemically measure the robustness of the input–output network using traditional methods. Thus, in this paper, we evaluate the robustness of the Chinese input–output network under random failure or intentional attack based on the relative entropy model.

## 2. Relative Entropy Model

### 2.1. Relative Entropy Theory

Relative entropy, known as Kullback–Leibler divergence (KLD), is a way of describing the difference between two probability distributions P and Q, shown in Equations (1) and (2). The former is the definition of relative entropy for the discrete random variables, and the latter is the definition for the continuous random variables.

(1) $$D(P|\left|Q\right)=\sum P\left(x\right)log\left(\frac{P\left(x\right)}{Q\left(x\right)}\right)$$

(2) $$D(P|\left|Q\right)=\int P\left(y\right)log\left(\frac{P\left(y\right)}{Q\left(y\right)}\right)d\left(y\right)$$

where the base is generally omitted, which is usually set to 2, *e*, or 10 if needed. As long as the base is greater than 1, the above equations can be used to calculate the relative entropy.

In the field of information, relative entropy is used to measure the number of bits required to encode the average sample from P using Q-based encoding. In particular, P is the real distribution of data and Q is the theoretical distribution, model distribution or approximate distribution of P. The distance between two random distributions can be measured by relative entropy. The more similar the two distributions are, the smaller the relative entropy is. As the difference between the two distributions increases, the relative entropy value also increases. Therefore, relative entropy can compare the similarity of the distributions of something, and evaluate the relative size of change.

### 2.2. Relative Entropy Model on the Network Robustness

Robustness is used to characterize the insensitivity of the control system to characteristic or parameter disturbance, that is, the anti-interference ability of the system. In this paper, the robustness is used to characterize the degree of the structural characteristic changes in the input–output network under random failure or intentional attack, namely, network anti-interference.

As for the types of attack, complex networks are generally under random failure or intentional attack [18,20,29]. In this paper, the distribution range [*L*_min_, *L*_max_] of the relevant indicators of each node is divided into *m* segments. The probability of the relevant indicators of each node falling in each interval is P(*x_i_*) = $p_{i}$ (*i* = 1, 2, …, *m*) before random failure or intentional attack, Q(*x_i_*) = $q_{i}$ (*i* = 1, 2, …, *m*) after random failure and R(*x_i_*) = $r_{i}$ (*i* = 1, 2, …, *m*) after intentional attack. Thus, Equation (1) for the discrete random variables is adopted to calculate the relative entropy.

## 3. Relevant Indicators of the Input–Output Network

### 3.1. Node Degree Distribution

For the input–output network, *G* = (*N*, *E*), where *N* is the number of nodes, *E* is the number of connected edges, and node degree is the number of edges connecting to a node. For a directional and weighted network, the weighted degree is the sum of the weighted indegree and the weighted outdegree,

(3) $$D_{i}=\sum_{j=1}^{N}e_{ij}+\sum_{j=1}^{N}e_{ji},\;i\;\neq \;j$$

where $D_{i}$ is the weighted degree, $e_{ij}$ is the weight of edges of node *i* pointing to node *j*, and $e_{ji}$ is the weight of edges of node *j* pointing to node *i*.

Once the network is under attack and nodes or edges are deleted, the weighted degree of each node will change, and the relative size of the change can be used as an indicator to measure the robustness of the input–output network.

### 3.2. Centrality Based on the Strongest Path (SP)

Different from the traditional concept of the shortest path, which is the path connecting two particular nodes in the network with the least number of steps among all possible paths, Xu and Liang [5] put forward the concept of the SP rooted from SPA in the IO model, and redefined three new concepts of centrality: SP betweenness, downstream closeness and upstream closeness.

#### 3.2.1. Strongest Path

Structural path analysis (SPA) is a method to find supply chain paths that contribute most to a particular consumption-based account (CBA). In particular, to supply the production of sector *j*, there are multiple paths originating from all other sectors in the economy. Contributions of particular paths to the unitary output of sector *j* can be measured using the Taylor expansion of the Leontief inverse matrix [30].

The SP can be defined as a particular path that contributes the most to the unitary output of industry *j* among all possible paths from industry *i* to industry *j*, which represents the most important path of all possible paths of supply from one particular industry to another. The strength of a particular SP from industry *i* to industry *j* is measured as

(4) $$q_{ij}=\prod a_{ik_{1}}a_{k_{1}k_{2}}\ldots a_{k_{m}j}$$

where $a_{ij}$ indicates the technical coefficient, and the SP from industry *i* to *j* is identified as *i*→$k_{1}$→……→$k_{m}$→*j*.

#### 3.2.2. SP Betweenness

The SP betweenness of nodes or links indicates their ability as centers of transforming resources from all over the economic system into finished products to supply the whole economy. For a particular industry, the SP betweenness is defined as the weighted sum of strengths of all SPs in the IO network passing through it, not including those SPs that start or end at it:

(5) $$b_{i}=\Sigma_{s=1,s\neq i}^{n}\sum_{t=1,\;t\neq i}^{n}X_{t}q_{st}$$

Similarly, the SP betweenness for a particular link *i*→*j* (all of which can be coalesced into the SP matrix) is

(6) $$b_{i\to j\;}=\Sigma_{s=1}^{n}\sum_{t=1}^{n}X_{t}q_{st}$$

#### 3.2.3. Downstream Closeness and Upstream Closeness

Closeness in network analysis measures how far a particular node is to all other nodes based on their shortest paths. In IO networks, two SP-based closeness measures are defined including downstream closeness and upstream closeness. The former represents an important role as the supplier to downstream industries, has the ability to drive economic development, and is the average value of all SPs starting from a particular industry *i*:

(7) $$C_{\dot{i}}^{D}=\frac{1}{n-1}\sum_{j=1}^{n}X_{j}q_{ij}$$

Similarly, the latter represents the important role as the consumer of downstream industries, drives economic development, and is defined as the average value of all SPs ending at a particular industry *j*:

(8) $$C_{\dot{j}}^{U}=\frac{1}{n-1}X_{j}\sum_{j=1}^{n}q_{ij}$$

where $X_{t}$ and $X_{j}$ represent the output of industries *i* and *j*, respectively.

Once the input–output network is under attack and node or edge fails occur, the value of SP betweenness, downstream closeness and upstream closeness will change. The magnitude of the change can be used to measure the robustness of the network.

## 4. Experiment

### 4.1. Chinese Input–Output Network Model

**Figure 1:**
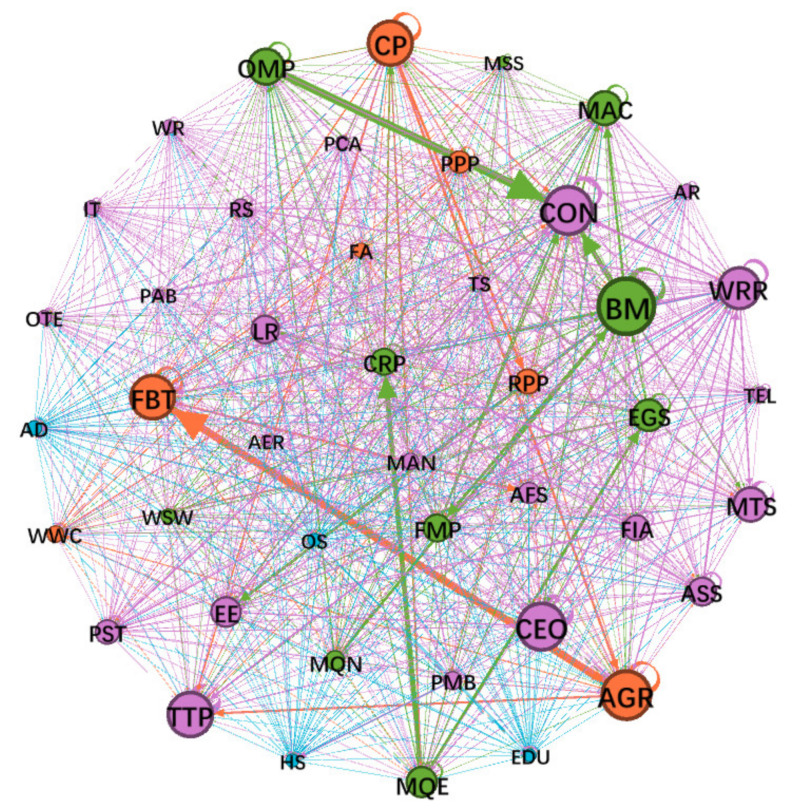
The Chinese input–output network model in 2018 (size per node indicates the weighted degree and width per edge indicates the edge weight).

The Chinese input–output network model (Figure 1) was built by the Chinese input–output table for the year 2018 from the most recent OECD input–output database (2021 edition, https://www.oecd.org/, accessed on 26 December 2021), which has 45 unique industries based on ISIC Revision 4 (while modeling, the 45th industry is deleted because all its data are zero). A list of OECD industries and corresponding abbreviations are in Appendix A Table A1. We will replace the full names of these industries with corresponding abbreviations in the following paragraphs.

Table 1 shows the node degree, SP betweenness, downstream closeness and upstream closeness of all the industries in the Chinese input–output network. It can be seen that the relative ordering of the four kinds of parameters is different. The top five industries by degree are the basic metals industry, the agriculture, hunting, and forestry industry, the construction industry, the computer, electronic and optical equipment industry and the textiles, textile products, leather and footwear industry, indicating the strength of connections between them and other nodes (industries). The top five industries by SP betweenness are the mining, quarrying, and energy-producing products industry, the basic metals industry, the coke and refined petroleum products industry, the food products, beverages and tobacco industry and the agriculture, hunting and forestry industry, indicating their ability to transform resources from all over the economic system into finished products to supply the whole economy. The top five industries by downstream closeness are the wholesale and retail trade and motor vehicle repair industry, the basic metals industry, the agriculture, hunting and forestry industry, the mining, quarrying, and energy-producing products industry and the chemical and chemical products industry, indicating their important role as suppliers to downstream industries and their ability to drive economic development. The top five industries by upstream closeness are the construction industry, the food products, beverages and tobacco industry, the basic metals industry, the machinery and equipment NEC industry and the wholesale and retail trade and motor vehicle repair industry, indicating their important role as consumers of downstream industries that then drive economic development.

### 4.2. Robustness Analysis

**Figure 2:**
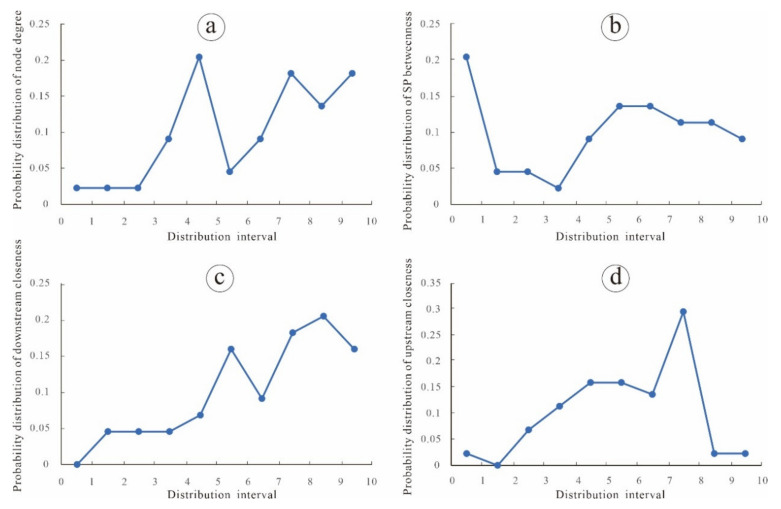
The original probability distribution of (**a**) node degree, (**b**) SP betweenness, (**c**) downstream closeness and (**d**) upstream closeness.

Complex networks usually face two types of attack: random failure and intentional attack. Random failure means that nodes or edges are attacked randomly with a certain probability and then become invalid. Intentional attack means that nodes or edges are attacked according to certain strategies and then become invalid. In this paper, the distribution range of the network parameters of each node is divided into 10 segments, and the original probability falling in each interval is P(*x_i_*) = $p_{i}$ (*i* = 1, 2,…, 10), before random failure or intentional attack (Table 1, Figure 2). The subsequent probability falling into each interval is Q(*x_i_*) = $q_{i}$ (*i* = 1, 2,…, 10) after random failure, and R(*x_i_*) = $r_{i}$ (*i* = 1, 2,…, 10) after intentional attack. After each attack, we can calculate the relative entropy according to the subsequent probability distribution of the network parameters and the original probability distribution.

#### 4.2.1. Node Attack

**Figure 3:**
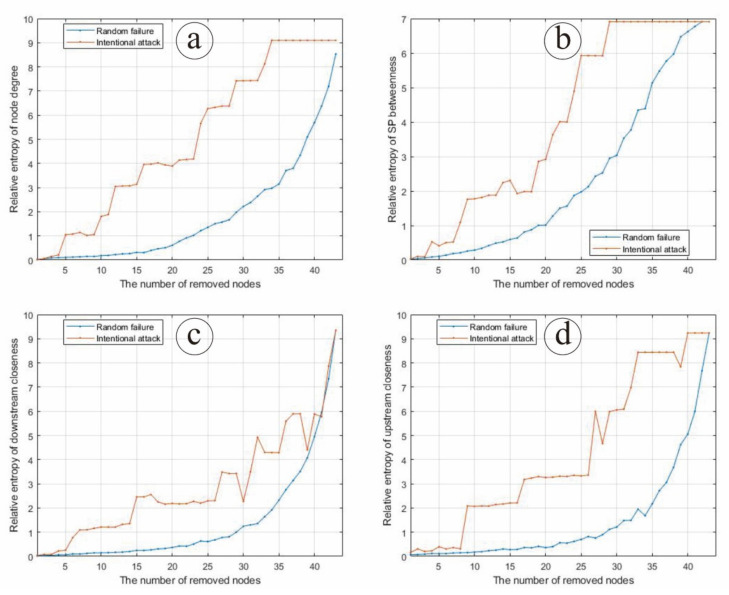
The changing situation of entropy under node random failure and intentional attack according to (**a**) node degree, (**b**) SP betweenness, (**c**) downstream closeness and (**d**) upstream closeness.

In the case of random node failure, nodes are deleted randomly in corresponding proportions, and then the average value of node degree, SP betweenness, downstream closeness and upstream closeness are calculated with 100 simulations, respectively. In the case of intentional node attack, nodes are deleted in corresponding proportions according to node degree, SP betweenness, downstream closeness and upstream closeness, respectively, and then the probability distributions of three network parameters (node degree, node clustering coefficient and intermediary centrality under the strongest path) are calculated. Based on the relative entropy theory, the relative entropy of the probability distribution of the relevant parameters before and after random attack and intentional attack of the Chinese input–output network are calculated, respectively (Figure 3).

As can be seen from Figure 3a, the relative entropy of the node degree distribution of the Chinese input–output network gradually increases with the increase in the proportion of nodes under random failure and intentional attack. Under random node failure, the relative entropy of node degree distribution increases slowly. Under intentional node attack, the relative entropy of node degree distribution increases rapidly, and remains stable when the number of nodes exceeds 33. When the Chinese input–output network is under intentional attack, the relative entropy of node degree distribution is always larger than when the Chinese input–output network is under random failure, indicating that intentional node attack on the node degree distribution of the Chinese input–output network may make an even stronger impact than random node failure.

As can be seen from Figure 3b, the relative entropy of SP betweenness distribution of the Chinese input–output network gradually increases with the increase in the proportion of nodes under random failure and intentional attack, which is similar to node degree. Under random node failure, the relative entropy of SP betweenness distribution increases slowly. Under intentional node attack, the relative entropy of SP betweenness distribution increases rapidly, and remains stable when the number of nodes exceeds 29. When the Chinese input–output network is under intentional attack, the relative entropy of SP betweenness distribution is always larger than when the Chinese input–output network is under random failure, indicating that intentional node attack on the SP betweenness of the Chinese input–output network may make an even stronger impact than random node failure.

As can be seen from Figure 3c, the relative entropy of the downstream closeness distribution of the Chinese input–output network gradually increases with the increase in the proportion of nodes under random failure and intentional attack. Under random node failure, the relative entropy of downstream closeness distribution increases slowly at first and rapidly afterwards. Under intentional node attack, the relative entropy of downstream closeness distribution increases with a circle variation, and tends to coincide with that of random failure when the number of nodes exceeds 40. When the Chinese input–output network is under intentional attack, the relative entropy of downstream closeness distribution is always larger than when the Chinese input–output network is under random failure, indicating that intentional node attack on the downstream closeness of the Chinese input–output network may make an even stronger impact than random node failure.

As can be seen from Figure 3d, the relative entropy of the upstream closeness distribution of the Chinese input–output network gradually increases with the increase in the proportion of nodes under random failure and intentional attack. When the Chinese input–output network is under intentional attack, the relative entropy of upstream closeness distribution increases with a circle variation, which is similar to downstream closeness, and it is always larger than when the Chinese input–output network is under random failure, indicating that intentional node attack on the upstream closeness of the Chinese input–output network may make an even stronger impact than random node failure.

In general, when the Chinese input–output network is under intentional node attack, the relative entropy of network node parameters is higher than that under random node attack, and increases faster. Therefore, when the Chinese input–output network is under intentional node attack, its structure and function change greatly, that is, the robustness is weak. When subjected to random node failure, the economic structural characteristics remain good within a certain range, and the damage degree of the structure shows a slow trend, which indicates that the robustness is strong.

#### 4.2.2. Edge Attack

The SP betweenness for links (namely, the SP matrix) between all industries in the Chinese input–output network using Equations (6) and (8) can also reflect the ability to transform resources.

**Figure 4:**
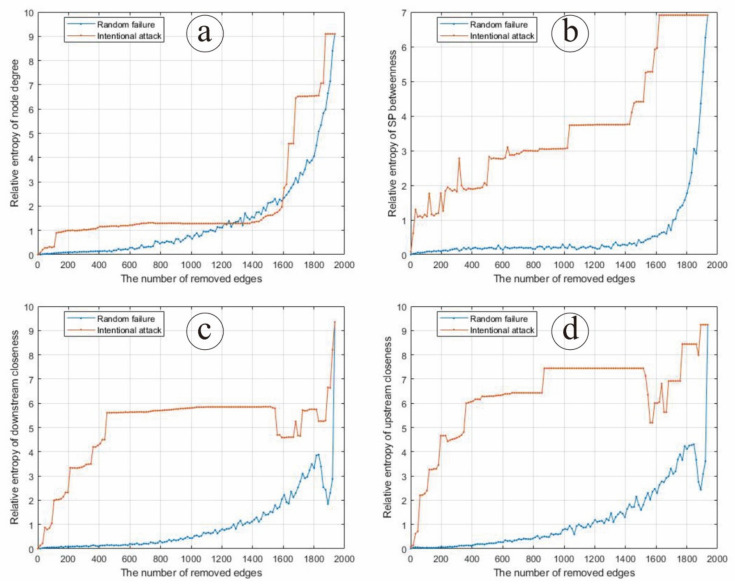
The changing situation of entropy under edge random failure and intentional attack according to (**a**) node degree, (**b**) SP betweenness, (**c**) downstream closeness and (**d**) upstream closeness.As can be seen from Figure 4a, the relative entropy of node degree distribution of the Chinese input–output network gradually increases slowly at first and quickly afterwards, with an increase in the proportion of edges under random failure and intentional attack. Overall, the relative entropy of node degree distribution under intentional edge attack is slightly larger than under random edge attack, except when the 1300th–1600th edges are under attack, indicating that an intentional edge attack on the node degree of the Chinese input–output network may make an even stronger impact than random edge failure.

In the case of intentional edge attack, we delete the corresponding edges in the input–output matrix according to the data size of the SP matrix, and then calculate the node probability distribution of four types of network parameters (node degree, SP between, downstream closeness and upstream closeness), respectively. In the case of random edge failure, we randomly delete the edges in corresponding proportion, run 100 simulations to calculate the average value of node degree, SP betweenness, downstream closeness and upstream closeness, respectively, and then calculate the probability distribution of three network parameters (node degree, node clustering coefficient and intermediary centrality under the strongest path). Based on the relative entropy theory, the relative entropy of the probability distribution of relevant parameters before and after random attack and the intentional attack of the Chinese input–output network is calculated, respectively (Figure 4).

As can be seen from Figure 4b, the relative entropy of SP betweenness distribution of the Chinese input–output network gradually increases with the increase in the proportion of edges under random failure and intentional attack. The relative entropy of SP betweenness distribution under random edge failure increases with a circle variation, and is apparently higher than that under random edge failure, indicating that the intentional edge attack on SP betweenness of the Chinese input–output network may make an even stronger impact than random edge failure.

As can be seen from Figure 4c, the relative entropy of downstream closeness distribution of the Chinese input–output network gradually increases with a circle variation with an increase in the proportion of edges under random failure and intentional attack. The relative entropy of downstream closeness distribution hits a plateau when the 400th–1600th edges are under intentional attack, and then increases quickly. Overall, when the Chinese input–output network is under intentional edge attack, the relative entropy of downstream closeness distribution is always larger than when the Chinese input–output network is under random edge failure, indicating that intentional edge attack on the downstream closeness of the Chinese input–output network may make an even stronger impact than random edge failure.

As can be seen from Figure 4d, the relative entropy of the upstream closeness distribution of the Chinese input–output network gradually increases with the increase in the proportion of edges under random failure and intentional attack. The relative entropy of upstream closeness distribution hits a plateau when the 400th–1600th edges are under intentional attack, and then increases quickly, which is quite similar to that of downstream closeness. When the Chinese input–output network is under intentional edge attack, the relative entropy of upstream closeness distribution is always larger than that under random edge failure, indicating that intentional edge attack on the upstream closeness of the Chinese input–output network may make an even stronger impact than random edge failure.

In general, when the Chinese input–output network is under intentional edge attack, the relative entropy of the network node parameters is higher than that under random edge attack, and increases faster. Therefore, when the Chinese input–output network is under intentional edge attack, its structure and function change greatly, that is, the robustness is weak. When subjected to random edge failure, the economic structural characteristics remain good within a certain range, and the damage degree of the structure shows a slow trend, which indicates that the robustness is strong.

## 5. Conclusions

(1) The relative entropy of network node parameters (node degree, SP betweenness, downstream closeness and upstream closeness) is relatively large, and increases relatively quickly when the Chinese input–output network is under intentional node or edge attack, indicating strong robustness.

(2) The relative entropy of network node parameters (node degree, SP betweenness, downstream closeness and upstream closeness) is relatively small, and increases relatively slowly when the Chinese input–output network is under random node or edge failure, indicating weak robustness.

(3) Meanwhile, our experiments show that the relative entropy model is applicative and effective in measuring the robustness of the IO network.

## Figures and Tables

**Table 1:** Relevant parameters (node degree, SP betweenness, downstream closeness and upstream closeness) in the Chinese input–output network.

Serial Number	Industrial Abbreviation	Weighted Degree	SP Betweenness	Downstream Closeness	Upstream Closeness
1	AGR	2,562,629	92,472	26,293	16,338
2	FA	256,312	2461	3340	2338
3	MQE	1,339,448	245,831	24,536	7366
4	MQN	587,029	8003	10,456	4708
5	MSS	49,156	0	1724	505
6	FBT	2,255,791	131,243	15,269	25,130
7	TTP	2,297,492	711	6788	12,821
8	WWC	415,010	2724	4125	2791
9	PPP	789,608	7407	7092	4918
10	CRP	1,069,326	152,486	14,323	13,828
11	CCP	2,283,824	85,214	23,024	13,646
12	PMB	517,523	26,165	3523	4108
13	RPP	969,176	19,281	9262	8350
14	OMP	1,713,398	89,729	18,940	11,706
15	BM	3,072,146	185,195	30,339	18,371
16	FMP	1,142,253	13,689	11,234	11,366
17	CEO	2,471,357	3147	8713	14,210
18	EE	1,301,461	23,716	10,668	14,316
19	MAC	1,578,061	7072	9637	17,375
20	MTS	1,596,877	16,268	6142	12,870
21	OTE	256,789	4996	1272	3352
22	MAN	392,986	9027	2860	5779
23	EGS	1,443,474	21,102	13,240	11,682
24	WSW	257,270	211	2869	2204
25	CON	2,516,932	4015	676	55,852
26	WRR	2,082,205	65,849	31,445	16,458
27	LR	1,171,919	64,100	15,539	11,879
28	WR	206,296	2067	2490	2408
29	AR	204,366	1227	2582	1987
30	TS	258,371	0	3526	2831
31	PCA	165,302	127	2257	1390
32	AFS	708,324	40,696	7459	9312
33	PAB	70,715	0	606	981
34	TEL	299,942	0	2722	2831
35	IT	277,840	0	2850	2721
36	FIA	998,099	4019	18,777	3434
37	RS	489,535	82	7268	4375
38	PST	911,260	3791	11,206	8695
39	ASS	1,190,835	6898	14,674	10,311
40	PD	282,407	0	251	6113
41	EDU	170,692	0	357	3498
42	HS	177,786	354	192	4433
43	AER	79,489	0	410	994
44	OS	176,348	0	1752	2129

## Appendix A

**Table A1:** List of OECD industries and corresponding abbreviations.

Serial Number	ISIC Rev.4	Industry	Abbreviation
1	D01T02	Agriculture, hunting, forestry	AGR
2	D03	Fishing and aquaculture	FA
3	D05T06	Mining and quarrying, energy producing products	MQE
4	D07T08	Mining and quarrying, non-energy producing products	MQN
5	D09	Mining support service activities	MSS
6	D10T12	Food products, beverages and tobacco	FBT
7	D13T15	Textiles, textile products, leather and footwear	TTP
8	D16	Wood and products of wood and cork	WWC
9	D17T18	Paper products and printing	PPP
10	D19	Coke and refined petroleum products	CRP
11	D20	Chemical and chemical products	CCP
12	D21	Pharmaceuticals, medicinal chemical and botanical products	PMB
13	D22	Rubber and plastics products	RPP
14	D23	Other non-metallic mineral products	OMP
15	D24	Basic metals	BM
16	D25	Fabricated metal products	FMP
17	D26	Computer, electronic and optical equipment	CEO
18	D27	Electrical equipment	EE
19	D28	Machinery and equipment, nec	MAC
20	D29	Motor vehicles, trailers and semi-trailers	MTS
21	D30	Other transport equipment	OTE
22	D31T33	Manufacturing nec; repair and installation of machinery and equipment	MAN
23	D35	Electricity, gas, steam and air conditioning supply	EGS
24	D36T39	Water supply; sewerage, waste management and remediation activities	WSW
25	D41T43	Construction	CON
26	D45T47	Wholesale and retail trade; repair of motor vehicles	WRR
27	D49	Land transport and transport via pipelines	LR
28	D50	Water transport	WR
29	D51	Air transport	AR
30	D52	Warehousing and support activities for transportation	TS
31	D53	Postal and courier activities	PCA
32	D55T56	Accommodation and food service activities	AFS
33	D58T60	Publishing, audiovisual and broadcasting activities	PAB
34	D61	Telecommunications	TEL
35	D62T63	IT and other information services	IT
36	D64T66	Financial and insurance activities	FIA
37	D68	Real estate activities	RS
38	D69T75	Professional, scientific and technical activities	PST
39	D77T82	Administrative and support services	ASS
40	D84	Public administration and defence; compulsory social security	PD
41	D85	Education	EDU
42	D86T88	Human health and social work activities	HS
43	D90T93	Arts, entertainment and recreation	AER
44	D94T96	Other service activities	OS
45	D97T98	Activities of households as employers; undifferentiated goods- and services-producing activities of households for own use	HOU
